# The LPS Responsiveness in BN and LEW Rats and Its Severity Are Modulated by the Liver

**DOI:** 10.1155/2018/6328713

**Published:** 2018-07-30

**Authors:** Haoshu Fang, Hao Jin, Chuanfeng Hua, Anding Liu, Zichen Song, Xulin Chen, Olaf Dirsch, Uta Dahmen

**Affiliations:** ^1^Department of Pathophysiology, Anhui Medical University, Hefei 230032, China; ^2^Experimental Transplantation Surgery, Department of General, Visceral and Vascular Surgery, Jena University Hospital, 07747 Jena, Germany; ^3^Transplantation Center, First Affiliated Hospital of Wenzhou Medical University, Wenzhou, Zhejiang 325000, China; ^4^Experimental Medicine Center, Tongji Hospital, Tongji Medical College, Huazhong University of Science and Technology, Wuhan 430030, China; ^5^Department of Burns, The First Affiliated Hospital of Anhui Medical University, Hefei 230022, China; ^6^Institute of Pathology, Hospital of Chemnitz, 09131 Chemnitz, Germany

## Abstract

Differences in LPS responsiveness influence the outcome of patients with sepsis. The intensity of the response is highly variable in patients and strain dependent in rodents. However, the role of the liver for initiating the LPS response remains ill defined. We hypothesize that hepatic LPS uptake is a key event for initiating the LPS response. In the present study, the severity of the LPS-induced inflammatory response and the hepatic LPS uptake was compared in two rat strains (Lewis (LEW) rats and Brown Norway (BN) rats). Using a transplantation model, we demonstrated the decisive role of the liver. The expression of hepatic TNF-*α*, IL-6, and IL-1*β* mRNA levels in BN rats was significantly lower than that in LEW rats. *LEW rats were sensitized* to LPS via G-CSF pretreatment. Sensitization caused by G-CSF pretreatment induced severe liver injury and mortality in LEW rats, but not in BN rats (survival rate: 0% (LEW) versus 100% (BN), *p* < 0.01). LEW rats presented with higher liver enzymes, more alterations in histology, and higher expression of caspase 3 and higher cytokines levels. One of the reasons could be the increased hepatic LPS uptake, which was only observed in LEW but not in BN livers. Using the transplantation model revealed the decisive role of the LPS responsiveness of the liver. Injection of LPS to the high-responding LEW recipient before transplantation of a low-responder BN liver resulted in a 50% survival rate. In contrast, injecting the same dose of LPS into the high-responding LEW recipient after transplanting the low-responding BN liver resulted in a 100% survival rate. The severity of inflammatory response in different strains might be related to the differences in hepatic LPS uptake. This observation suggests that the liver plays a genetically defined decisive role in modulating the inflammatory severity.

## 1. Introduction

Liver dysfunction is one of the earliest high-risk factors for multiorgan failure in patients with sepsis [1]. However, liver dysfunction does not occur to the same extent in all patients. Susceptibility to lipopolysaccharide (LPS) and sepsis is highly variable. As the liver seems to play a pivotal role in metabolic and immunological homeostasis, alterations in its function can promote the progression of multiple organ failure [2].

The differences in the severity of hepatic dysfunction in septic patients might be caused by differences in the LPS responsiveness [3, 4]. The liver is the main organ that clears LPS. Growing evidence has been accumulated that the liver plays a major role in both hepatic local and systemic inflammatory response [5–7]. The liver is involved in the inflammatory response via the production of inflammatory cytokines and complement factors [7]. During the inflammatory response, the liver-derived proinflammatory cytokines, such as TNF-*α*, IL-6, and IL-1, can activate the acute phase response by initiating the production of further acute-phase proteins, thereby combating local and systemic pathogen infection.

LPS is considered to be the most important bacterial factor in the pathogenesis of systemic inflammatory response syndrome (SIRS) [8, 9]. LPS leads to a dose-dependent inflammatory response of the organism, ultimately resulting in the SIRS, multiorgan failure, and death of the organism. Attempts to define the concordance between gram-negative bacteremia and endotoxemia in patients with sepsis have been controversial [10]. Of course, part of the reason is due to the limitations of the detection method (e.g., limulus test) and the high number of structural variations of LPS, due to the high number of different gram-negative bacterial strains of origin. However, whether these elusive observations were also related to the differences in LPS responsiveness of individuals remains largely unknown.

Our previous results indicated that the LPS sensitivity could be induced by granulocyte colony-stimulating factor (G-CSF) pretreatment [9]. Using this pretreatment, the severity of the inflammatory response and the subsequent inflammatory injury to the same dose of LPS could be modulated. However, the role of the liver in the modulation of LPS-induced inflammatory response remains undefined.

In the present study, we hypothesize that the severity of LPS-induced inflammatory response was mainly modulated in the liver via the extent of hepatic LPS uptake. In order to investigate this hypothesis, we observed the severity of the inflammatory response and LPS uptake in naïve and sensitized rats of two strains (Lewis (LEW) rats and Brown Norway (BN) rats) with different sensitivities to LPS. Furthermore, we transplanted a low-responder liver into a high-responder recipient and treated the recipient either before (to BN rats) or after the transplantation (to LEW rats) with a single sublethal dose of LPS.

## 2. Materials and Methods

### 2.1. Experimental Design

The inflammatory response was provoked by the administration of a sublethal dose of LPS (2 mg/kg, intravenous injection; *E. coli* serotype O55:B05 type, Sigma-Aldrich, St. Louis, USA) to LEW and BN rats (Figure S1A).

LPS sensitization was induced by pretreating the rats with G-CSF (100 *μ*g/kg/day for 5 days, subcutaneous injection, Ratiopharm, Breda, Netherlands) prior to LPS administration as described before [9] (Figure S1B).

In order to confirm that LPS susceptibility was liver dependent, BN-to-LEW orthotopical liver transplantation (LT) was performed. LPS was injected either immediately before or after transplantation to LEW donors with the LEW liver or after transplantation to LEW donors with the BN liver (Figure S1).

### 2.2. Animals

Male LEW and BN rats (14 weeks old, body weight 300 ± 50 g) were purchased from Charles River Laboratories (Sulzfeld, Germany). The animals were housed under standard animal care conditions and had free access to water and rat chow ad libitum. Animals were allowed to adapt to laboratory conditions for at least seven days. All procedures and operations were performed according to the German Animal Welfare Legislation. Prior to surgery, inhalation anesthesia (3% isoflurane, Sigma Delta, London, UK) was started. The experimental protocols were approved by the Thüringer Landesamt für Verbrancherschutz, Thuringia, Germany (Project number 02-043/10).

### 2.3. Rat Orthotopic Liver Transplantation

The nonarterialized orthotopic rat LT procedure was performed following the method described by Kamada and Calne [11]. Briefly, in donor operation, the suprahepatic vena cava, infrahepatic vena cava, and portal vein were mobilized. The common bile duct was cannulated by using a 22-gauge cannula. After heparinization, the donor liver was flushed by 4°C saline. 14-gauge and 10-gauge catheters (Becton Dickinson Infusion Therapy Systems Inc., Sandy, UT, USA) were installed in the portal vein and the infrahepatic inferior vena cava. The suprahepatic vena cava was reconstructed using 7-0 polypropylene suture material (Resorbe, Nuremberg, Germany). The infrahepatic vena cava, bile duct, and portal vein were reconstructed.

### 2.4. In Vivo Modulation of Inflammatory Response

LPS sensitization was induced by G-CSF pretreatment as described above [9]. Rats were pretreated with G-CSF for 5 days and challenged with LPS at the 6th day.

### 2.5. Organ Injury

The serum alanine transaminase (AST) and alanine aminotransferase (ALT) levels were measured by using an automated chemical analyzer (Bayer Advia 1650, Leverkusen, Germany).

### 2.6. Histological Staining

Liver tissue was fixed for 24 h (4.5% buffered formalin) and embedded in paraffin. The slides (4 *μ*m) were cut and stained with hematoxylin-eosin following the standard protocol described before [12]. Slides were digitized by using a Hamamatsu slide scanner (Hamamatsu Electronic Press Co. Ltd., Iwata, Japan) and assessed by two experienced pathologist and scientist (ODI and FHA). Evaluation was performed according to a standardized semiquantitative scoring system as described before [9] (Figure S2).

### 2.7. LPS-IHC

The LPS staining was performed as described before [13]. Briefly, the antigen retrieval was performed in 10 mM citrate buffer (pH 6.0), followed by incubation with a polyclonal mouse anti-LPS antibody (1 : 100, Abcam, Cambridge, UK) for 15 min at room temperature. Signals were amplified by using the Dako CSAII system (Glostrup, Denmark).

### 2.8. Quantitative Polymerase Chain Reaction (PCR)

Total RNA was isolated from liver tissue using the RNeasy kit (Qiagen, Hilden, Germany) and quantified by using NanoDrop spectrophotometer (ND-100, PEQLAB, Erlangen, Germany). Complementary DNA was synthesized using 3 *μ*g RNA (First-Strand cDNA synthesis kit, Invitrogen, Carlsbad, USA). Ten micrograms of cDNA was used for quantitative PCR reaction (Brilliant qPCR Master Mix kit, Agilent, Santa Clara, USA). The sequences of primers and probes were as follows: IL-6: CCTGGAGTTTGTGAAGAACAACT and GGAAGTTGGGGTAGGAAGGA (Probe #106), TNF-*α*: TGAACTTCGGGGTGATCG and GGGCTTGTCACTCGAGTTTT (Probe #63), TLR4: GGATGATGCCTCTCTTGCAT and TGATCCATGCATTGGTAGGTAA (Probe #95), MD2: TGATGATTATTCTTTTTGCAGAGC and ATCCCCAGCAATGGCTTC (Probe #75), and hypoxanthine guanine phosphoribosyltransferase (HPRT): GACCGGTTCTGTCATGTCG and ACCTGGTTCATCATCACTAATCAC (Probe #95). The gene expression fold changes were calculated using pooled liver samples from all groups as the reference sample.

### 2.9. Enzyme-Linked Immunosorbent Assay (ELISA)

Serum TNF-*α*, IL-6, and IL-10 levels were determined with a commercial ELISA kit (R&D, Minneapolis, USA). All procedures were performed according to the instructions of the manufacturer. The signal was detected by using an SLT Spectra ELISA plate reader at 450 nm with the correction of 540 nm.

### 2.10. Western Blot

The total protein was isolated from liver tissues using RIPA buffer (Sigma-Aldrich, St. Louis, US). Ten micrograms of total protein was loaded per well and then separated on 12% sodium dodecyl sulfate-polyacrylamide gel. Then, the protein was transferred onto the polyvinylidene difluoride membrane (GE Healthcare, Buckinghamshire, United Kingdom). The membrane was blocked with 5% BSA in TBST (0.5% Tween 20) for 1 h at room temperature. The membrane was incubated at 4°C overnight with primary antibodies: rabbit anti-phospho-AKT (Ser473, 1 : 1000), rabbit anti-phospho-Stat3 (Tyr705, 1 : 2000), rabbit anti-phospho-NF-*κ*B p65 (Ser536, 1 : 1000), rabbit anti-AKT (1 : 1000), rabbit anti-Stat3 (1 : 2000), anti-NF-*κ*B p65 (1 : 1000), rabbit anti-caspase 3 (1 : 1000), and rabbit anti-GAPDH (1 : 20000, Sigma-Aldrich, St. Louis, USA). All antibodies besides rabbit anti-GAPDH were purchased from Cell Signaling Technology, Beverly, USA. The membrane was incubated for 1 h at room temperature with a goat polyclonal to rabbit IgG antibody (1 : 10000, Abcam) and developed with the SuperSignal West Femto Trial kit (Thermo, USA). The membrane was exposed to high-sensitivity films (GE Healthcare) for autoradiography.

### 2.11. Statistical Analysis

All data were expressed as mean ± SD. The statistical calculations were performed by using SigmaStat (ver. 3.5.54; Systat Software GmbH, Erkrath, Germany), and the figures were produced by using SigmaPlot (ver. 12.5; Systat Software GmbH, Erkrath, Germany). The survival rate was analyzed by performing a log-rank test. Values were compared by using the *t*-test in case of normal distribution of the data. If data were not normally distributed, the Mann–Whitney rank-sum test was used to compare sets of data in different animal groups. The survival rate was analyzed by using the log-rank method. *p* < 0.05 was considered statistically significant in this experiment.

## 3. Results

### 3.1. LPS Administration Induced a More Severe Inflammatory Response in LEW Rats than in BN Rats

**Figure 1:**
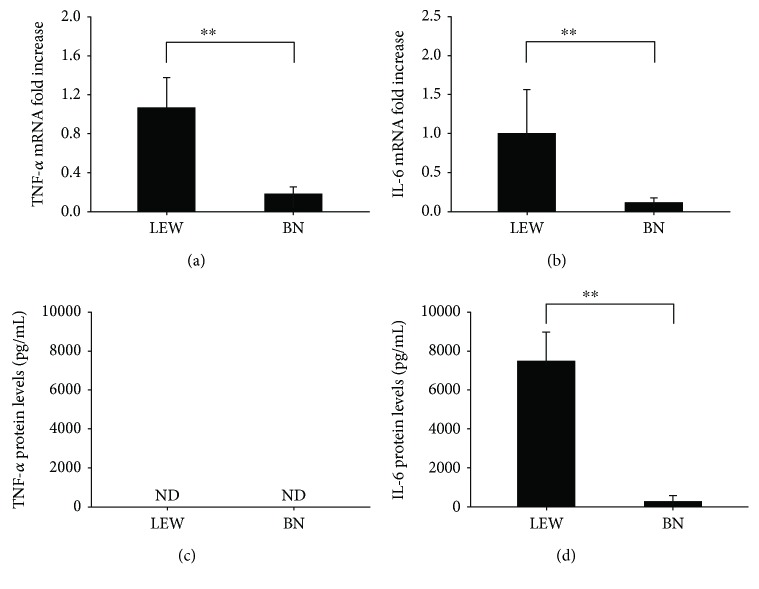
The LPS-induced inflammatory response was compared in LEW and BN rats. Hepatic TNF-*α* (a) and IL-6 (b) mRNA expression was measured from LEW and BN rats 6 h after LPS challenge. Serum TNF-*α* (c) and IL-6 (d) protein levels were measured from LEW and BN rats 6 h after LPS injection by using ELISA. Data were shown as mean ± SD (*n* = 6 per group); ^∗∗^ *p* < 0.01.

We observed significantly higher expression of hepatic TNF-*α* and IL-6 mRNA in LEW rats that in BN rats (Figures 1(a) and 1(b)). The elevated serum protein levels were observed in LEW rats, but not in BN rats (Figures 1(c) and 1(d)). However, the serum TNF-*α* was not detectable in both BN and LEW rats 6 h after LPS injection. This might be due to delayed observation time [8]. The hepatic expression of TLR4 and MD2 mRNA was also detected by RT-PCR, but the difference between the two groups cannot reach significance (Figure S3).

### 3.2. G-CSF Pretreatment Induced LPS Sensitization in LEW Rats but Not in BN Rats

**Figure 2:**
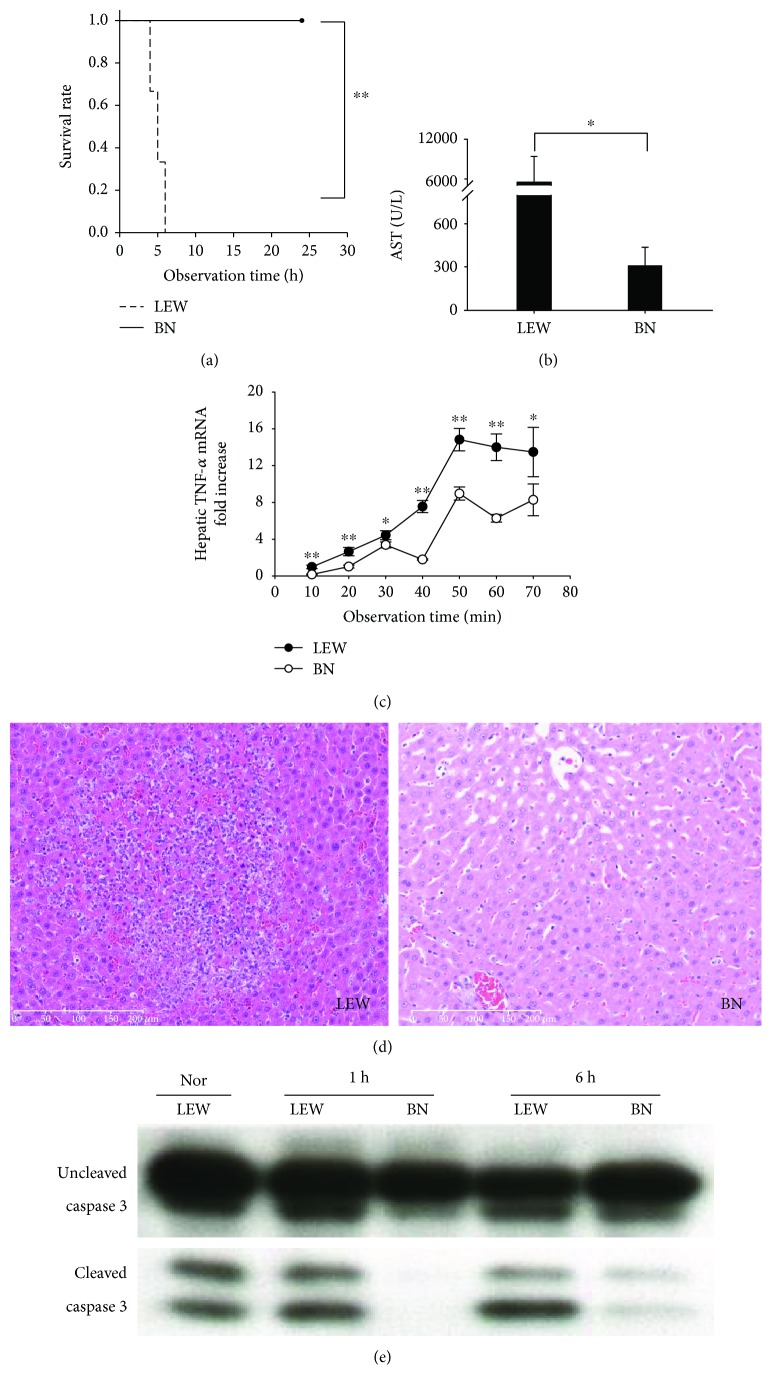
The G-CSF-induced LPS sensitization was strain dependent. (a) The survival rate after G-CSF pretreatment and LPS administration in LEW rats and BN rats, respectively. (b) The serum AST level was measured 6 h after G-CSF pretreatment and LPS administration. If the rats did not survive for 6 h, the blood samples were harvested at the time of death. (c) Hepatic TNF-*α* mRNA expression was measured in LEW and BN rats after G-CSF pretreatment and LPS challenge every 10 min within 70 min. Data were shown as mean ± SD (*n* = 6 per group). ^∗^ *p* < 0.05, ^∗∗^ *p* < 0.01 (d) The hepatic injury was evaluated by HE staining in LEW and BN rats after G-CSF pretreatment and LPS administration. Representative images from 6 rats per group were selected. (e) The apoptosis was measured by detecting the cleaved levels of caspase 3. The experiments were repeated 3 times, and 1 representative result is shown.

LPS sensitization caused the death of all LEW rats [9] within 6 h after an otherwise sublethal LPS dose but did not lead to the death of a single BN rat (survival rate: 0% (LEW) versus 100% (BN), Figure 2(a)). Six hours after LPS administration, hepatic injury as indicated by serum AST levels was more than 20-fold higher in LEW rats than in BN rats (Figure 2(b)). At the same observation time point, severe histological damage was observed in LEW rats such as intraparenchymal bleeding, lymphocyte infiltration in the sinusoid, and confluent necrosis. In contrast, only moderate morphological changes were observed in the liver tissues of BN rats, consisting of slight hepatic necrosis and moderate neutrophil infiltration (Figure 2(d)).

Similarly, the early inflammatory activation after LPS administration was more pronounced in LEW rats. Hepatic mRNA expression of TNF-*α* was significantly higher in LEW rats than in BN rats (Figure 2(c)). Furthermore, caspase 3 levels were much higher in LEW rats than in BN rats suggesting a higher level of apoptosis at time points, 1 h and 6 h after LPS injection (Figure 2(e)).

**Figure 3:**
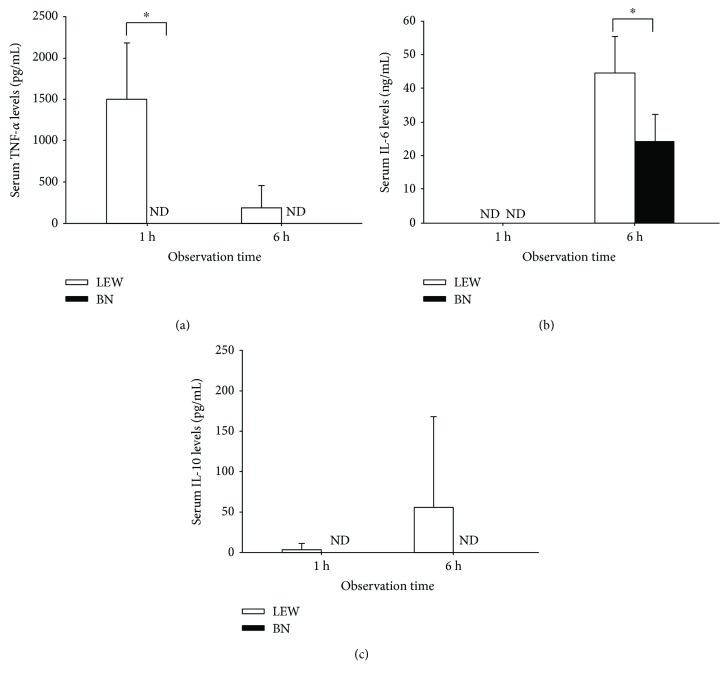
Systemic inflammatory response was compared in LEW and BN rats. The serum TNF-*α* (a), IL-6 (b), and IL-10 (c) levels were detected by using ELISA. ^∗^ *p* < 0.05, ND = not detected.

The induction of systemic inflammatory response was determined by the serum levels of inflammatory cytokines. As shown in Figure 3, G-CSF pretreatment and LPS administration caused the release of TNF-*α* in LEW rats. In contrast, TNF-*α* levels in BN rats remained below the detection levels despite the same treatment (Figure 3(a)). Moreover, 6 h after LPS administration, serum IL-6 levels were significantly higher in LEW rats than in BN rats (Figure 3(b)). Serum levels of IL-10 became detectable only in LEW rats, although in a rather low range, but not at all in BN rats (Figure 3(c)).

### 3.3. LPS Sensitization via G-CSF Pretreatment Caused Early Activation of Inflammation-Related Signal Pathways in LEW Rats but Rather Delayed Activation in BN Rats (Figure 4)

In LEW rats, LPS sensitization induced activation of p-p65, p-STAT3, and p-AKT which occurred as early as 1 h after LPS administration. In contrast, activation of these molecules was also induced in BN rats but delayed, indicated by increased phosphorylation levels of p-STAT3 and p-AKT occurring only at 6 hours. The delayed activation of signal pathways might lead to the observed decreased release of inflammatory cytokines, the lower apoptosis levels, and the rather discrete histological tissue injury.

### 3.4. LPS Sensitization Was Related to Hepatic LPS Uptake

**Figure 5:**
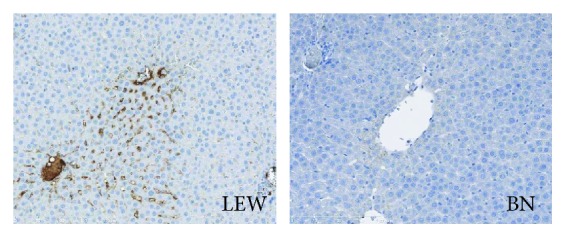
Hepatic LPS uptake was compared in LEW and BN rats. LPS IHC staining was performed to assess the hepatic uptake of LPS in LEW and BN rats 1 h after G-CSF pretreatment and LPS administration. Original magnification, ×200. Representative images from 6 rats per group were selected.

The severity of the inflammatory response was associated with the hepatic LPS uptake after G-CSF-induced LPS sensitization (Figure 5). LEW rats showed high intensity of LPS staining suggesting an intense hepatic uptake of LPS. In contrast, BN rats showed moderate intensity of hepatic LPS binding as visualized by IHC indicating a much lower hepatic LPS uptake. Therefore, we concluded that the strain difference in the LPS response of sensitized rats might be related to the hepatic LPS uptake.

### 3.5. The LPS-Induced Mortality Was Modulated upon Transplantation of a High-Responder Liver in a Low-Responder Organism

**Figure 6:**
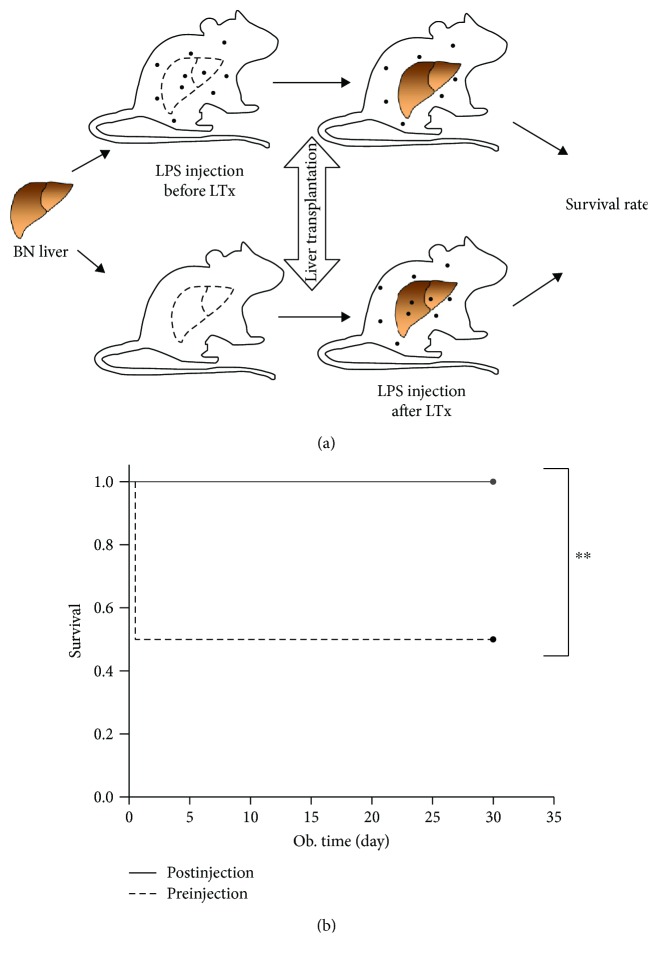
LPS-induced mortality was determined by hepatic LPS response in different strains. (a) Diagram of the experimental design. (b) Survival rate observed after LPS injection pre- and posttransplantation in the BN-to-LEW liver transplantation model. Replacements of a high-responder LEW liver with a low-responder BN liver reverse the mortality induced by LPS. *n* = 6 per group, ^∗∗^ *p* < 0.01.

LPS sensitivity in terms of LPS-induced mortality was modulated upon transplantation of a low-responder liver into a high-responder organism. Injection of LPS to the high-responding recipient before transplanting caused 50% mortality. In contrast, injecting LPS after transplanting the low-responding BN liver into the high-responding LEW recipient led to survival of all animals (Figure 6).

## 4. Discussion

In the present study, the LPS responsiveness was compared in LEW rats and BN rats. The LEW rats were more susceptible to LPS-induced inflammatory response than BN rats. Interestingly, this strain difference in inflammatory insult could be reversed by transplanting a low-responder BN liver to a high-responder LEW recipient.

### 4.1. Liver Is the Main Organ Modulating the Inflammatory Response

The LPS-induced inflammatory insult was reversed by transplanting a low-responder BN liver to a high-responder LEW recipient. Therefore, we now determined that the liver is important in the modulation of inflammatory response. The LPS-induced tissue damage occurred simultaneously in different organs and triggers multiorgan dysfunction [14]. Evidence revealed that the liver function might be critical to the prevention of other organ damage. Depleting the hepatic STAT3 and RelA ablated the acute-phase response induction and increased the mortality in a mouse pneumonia model [15]. The acute kidney failure is a common event in septic patients with liver cirrhosis [16]. These observations revealed that the liver is an important organ in mediating LPS-induced inflammatory response and triggering the systemic injury.

We observed that the hepatic LPS uptake was associated with the severity of inflammatory response. The different hepatic LPS uptake might lead to the activation of NF-*κ*B, STAT3, and AKT signal pathways and the induction of inflammatory response. Liu et al. [17] observed that blockade of hepatic LPS uptake significantly increased the survival rate in a rat sepsis model. Deng et al. reported that TLR4 on hepatocytes (HCTLR4) was required for efficient LPS clearance from circulation [18]. The depletion of HCTLR4 was associated with enhanced macrophage phagocytosis and improved survival rate in septic mice. Notably, the activation of the TLR4-NF-*κ*B signal axis after liver resection played a pivotal role in the LPS-induced liver failure and the mortality of the mice [19]. Knocking out the hepatic STAT3 in mice significantly reduced the circulating and air space acute-phase proteins and exhibited the elevated lung and blood bacterial burdens and mortality [20].

The LPS responsiveness might be individual dependent and influenced by several factors, including aging, drug treatment, and gene modification. The neonatal mice are more susceptible to various TLR stimuli and viral infection, which caused high mortality [21]. Treatment of alpha-galactosylceramide (*α*-GalCer) induced LPS sensitization and caused mortality of mice [22]. Our results demonstrated that the LEW rats were more sensitive to LPS insult than BN rats. The G-CSF pretreatment induced LPS sensitizations in LEW rats, indicated by severe inflammatory response, organ damage, and 100% mortality. However, the LPS sensitization was not observed in BN rats. It reported that these two rat strains are different with respect to the polarization of immune response and the susceptibility to autoimmune diseases, which was caused partly by the genetic variations [23]. These observations revealed the idea that the individuals' variable LPS responsiveness might be caused by the gene variation.

### 4.2. Clinically, the LPS Responsiveness Might Influence the Outcome of Patients with Sepsis and Infectious Disease

The severity of inflammatory response is variable among individuals [24]. The variation of inflammatory insult was observed in patients and associated with survival time [25, 26]. The heterogeneity in the observed severity of inflammatory response is partly caused by variations of the underlying genes leading to interindividual differences. The regulation of the inflammatory injury is influenced by the cytokines, chemokines, and regulatory proteins secreted by immune cells. TNF was considered an early mediator in the proinflammatory response. Tang et al. reported that the TNF gene variation was associated with sepsis severity and increased mortality of the patients after surgery [27]. The results from a meta-analysis indicated that the TNF-*α*-308 single-nucleotide polymorphism (SNP) results in the increased serum TNF levels and the increased risk for sepsis [28]. TNF-knockout mice exhibited reduced inflammatory response induced by LPS [29].

## 5. Conclusion

The severity of inflammatory response in different strains might be liver determined. These observations revealed that the liver plays a decisive role in the modulation of the inflammatory severity. Further investigation of the individuals' LPS responsiveness, especially studies focusing on the genetic variation related to LPS-induced inflammatory response, might be an effective way to treat infectious disease.

## Figures and Tables

**Figure 4:**
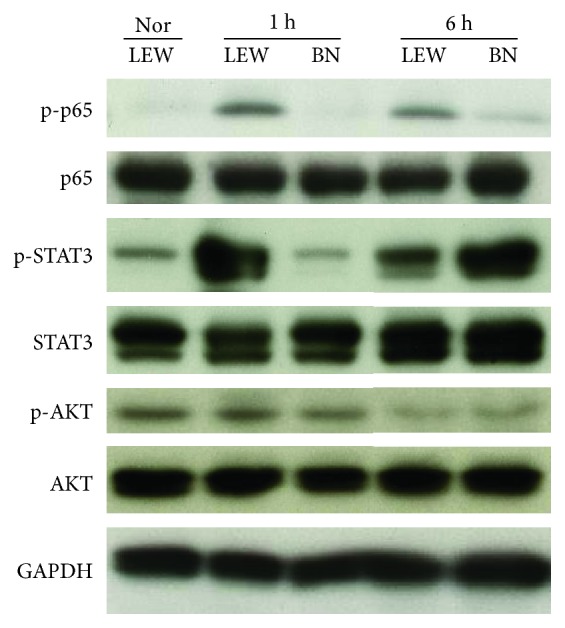
The activation of inflammatory response-related signal molecules was detected by performing Western blot. The phosphorylation and total levels of p65, STAT3, and AKT were detected by using protein samples isolated from rat liver tissues. The experiments were repeated 3 times, and 1 representative result is shown.

## Data Availability

Please contact the corresponding author for data requests.

## Supplementary Materials

Supplementary Materials Figure S1: experimental design to investigate the modulation of the LPS-induced inflammatory response. (A) The susceptibility to LPS-induced inflammatory response was examined in LEW and BN rats, (B) the sensitization of LPS-induced inflammatory response was examined by using G-CSF pretreatment, and (C) the susceptibility to LPS-induced inflammatory response was modulated by performing liver transplantation. Figure S2: parameters and morphology of the semiquantitative scoring system for histological evaluation. Figure S3: the expression of hepatic TLR4 and MD2 mRNA was compared in LEW and BN rats. The hepatic TLR4 (A) and MD2 (B) mRNA expression was detected by RT-PCR, and the difference in mRNA expression between the two groups cannot reach significance.
