# Supplementary material for: The LPS Responsiveness in BN and LEW Rats and Its Severity Are Modulated by the Liver
Source: J Immunol Res. 2018 Jul 30;2018:6328713. doi: 10.1155/2018/6328713 (PMC6091288; doi:10.1155/2018/6328713)

Figure S1. Experimental design to investigate modulation of the LPS induced inflammatory response. (A) The susceptibility of LPS induced inflammatory response was examined in Lew and BN rats, (B) the sensitization of LPS induced inflammatory response was examined by using G-CSF pretreatment, (C) the susceptibility of LPS induced inflammatory response was modulated by performing liver transplantation.


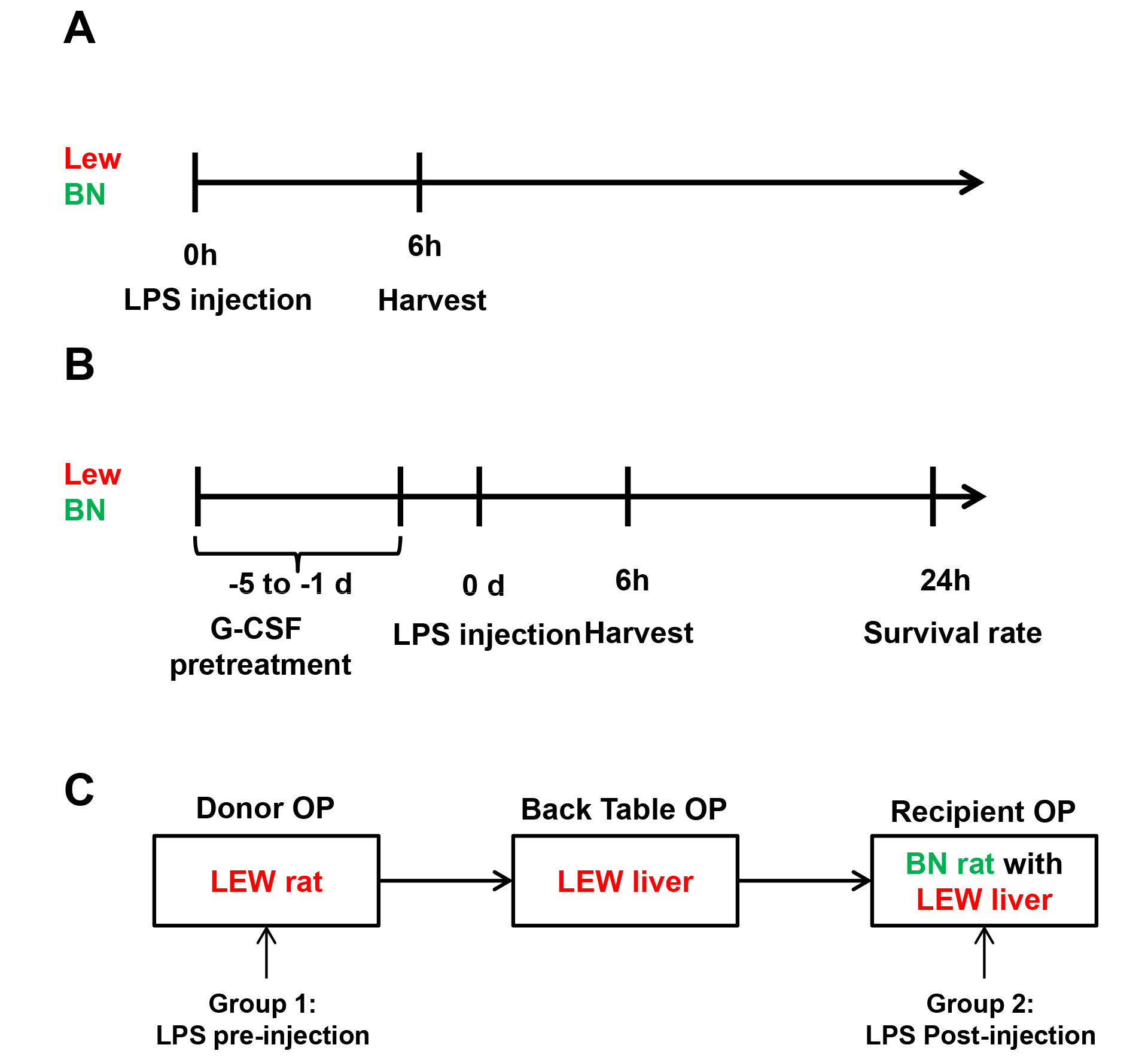


Figure S2. Parameters and morphology of semi-quantitative scoring system for histological evaluation.


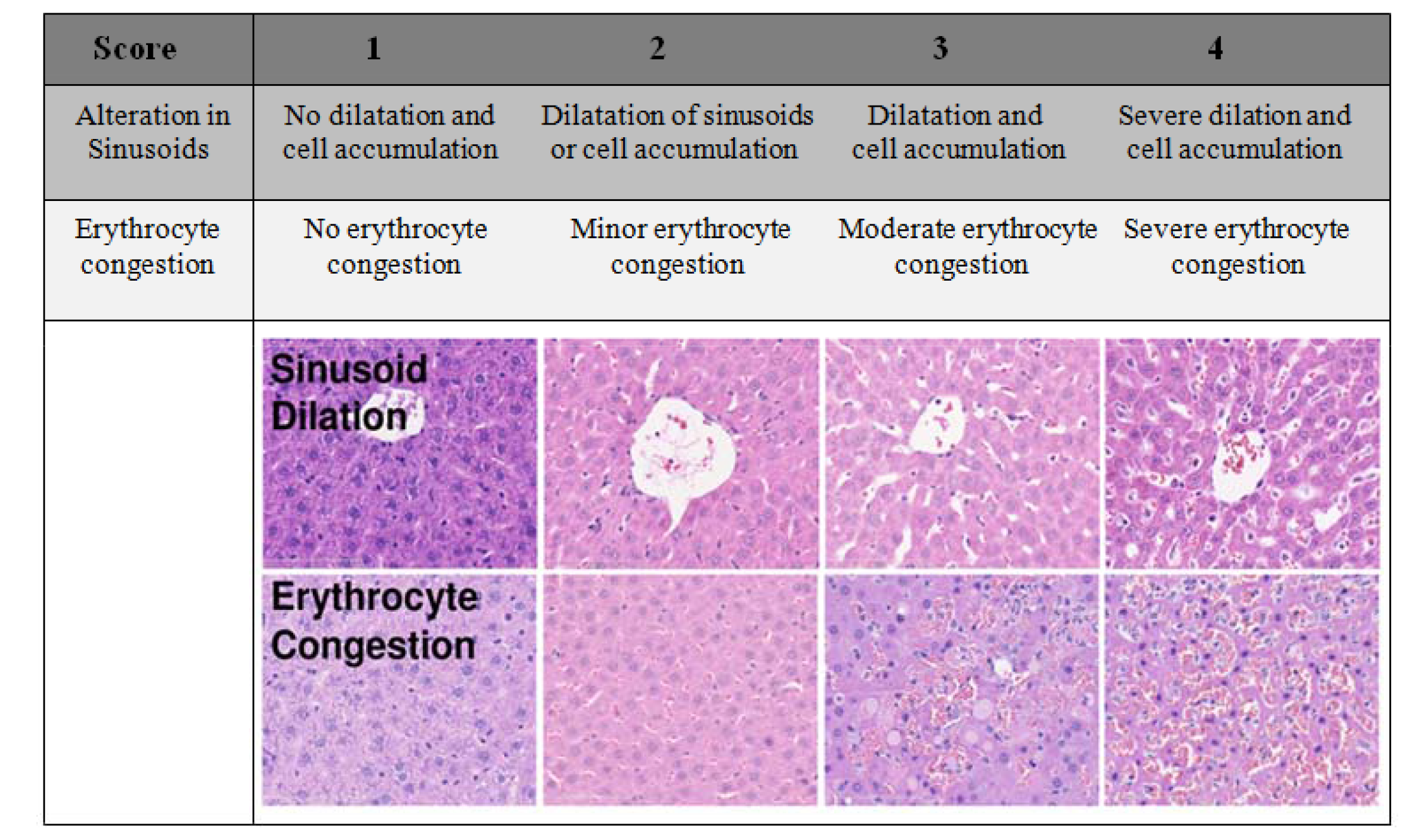


Figure S3. **The expression of hepatic TLR4, and MD2 mRNA was compared in LEW and BN rats.** The hepatic TLR4 (A), and MD2 mRNA expression (B) was detected by RT-PCR, the difference of mRNA expression between two groups cannot reach significant.


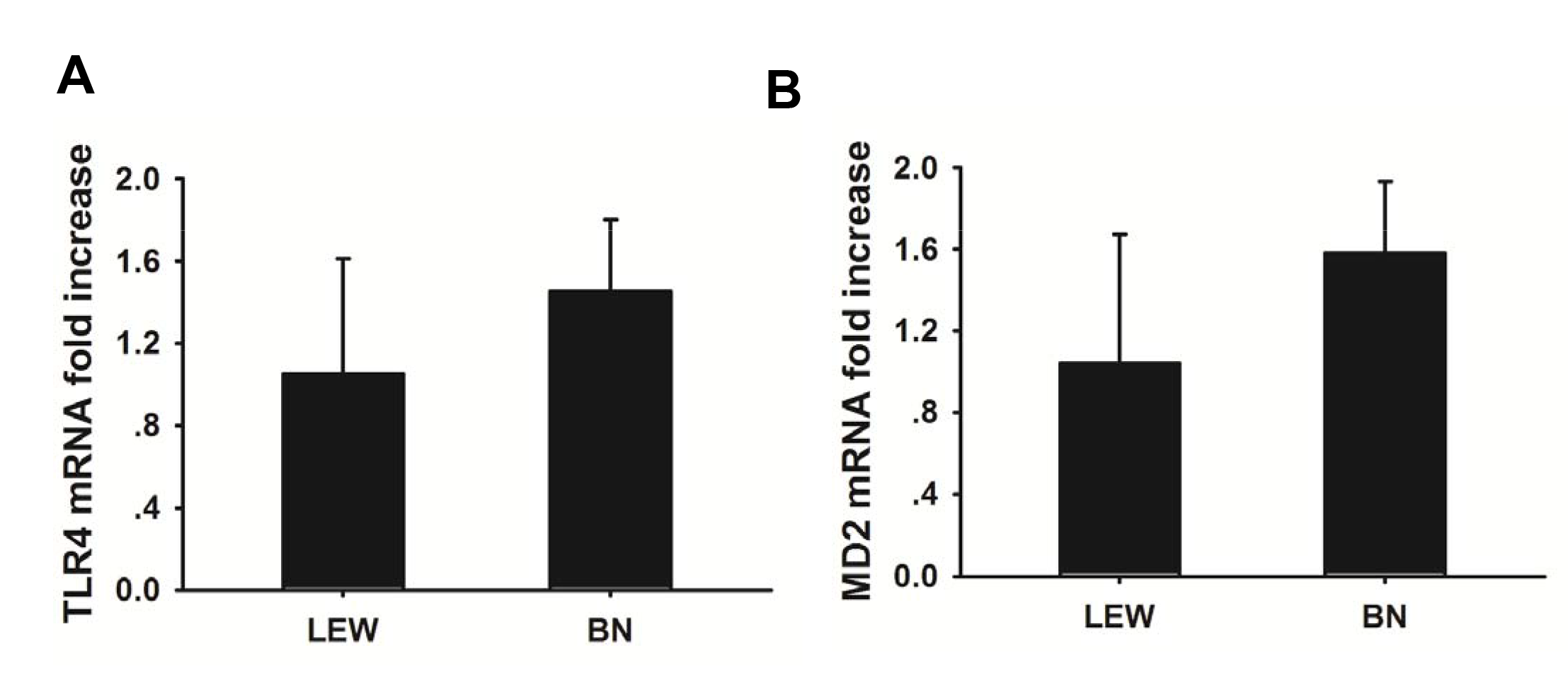

Supplement: Supplementary Materials — Figure S1: experimental design to investigate the modulation of the LPS-induced inflammatory response. (A) The susceptibility to LPS-induced inflammatory response was examined in LEW and BN rats, (B) the sensitization of LPS-induced inflammatory response was examined by using G-CSF pretreatment, and (C) the susceptibility to LPS-induced inflammatory response was modulated by performing liver transplantation. Figure S2: parameters and morphology of the semiquantitative scoring system for histological evaluation. Figure S3: the expression of hepatic TLR4 and MD2 mRNA was compared in LEW and BN rats. The hepatic TLR4 (A) and MD2 (B) mRNA expression was detected by RT-PCR, and the difference in mRNA expression between the two groups cannot reach significance. [file 6328713.f1.doc]
